# The Legal Principles Involved in Official Efforts for the Regulation or Suppression of Prostitution

**Published:** 1871-06

**Authors:** George Andrews

**Affiliations:** Attorney at Law; late Judge of the Supreme Court of Tennesee


### THE LEGAL PRINCIPLES INVOLVED IN OFFICIAL EFFORTS FOR THE REGULATION OR SUPPRESSION OF PROSTITUTION.

By GEORGE ANDREWS, Esq., Attorney at Law; late Judge of the Supreme Court of Tennessee.

Society has the right to protect itself, and to provide for the most perfect development of its members in body, intellect, and morals.

It may prohibit and punish every act found or decreed to be hurtful to the public welfare in either of these aspects.

To this end it may prohibit the injurious acts, not only where directed by force or violence against the body politic, or a member thereof, but where the act affects directly only the parties acting and consenting thereto, as in the case of gambling, and various forms of vice.

Society has a direct interest in the physical, mental, and moral perfection of its members, and may, without violating any natural right, so legislate as to advance, as far as possible, that perfection.

If those representing organized society as the law-making power, violate the trust reposed in them—if they legislate in malice or selfishness for private ends—if their legislation is found to be oppressive or injurious to the public welfare, and no other means to remedy the evil appears to the body of the society which they represent, there remains to the organized society, or the oppressed minority, the inalienable and sacred right of revolution. But, notwithstanding this ultimate right of rebellion or revolution, somewhere in every organized political society, exists legislative omnipotence. What legislation is in fact necessary or salutary, the organized society, or individual, or body, to whom the legislative power is by the organization of the particular society committed must determine, and there is no appeal from this decision but to the last refuge of the people against oppression.

In this sense, the Parliament of England, which is not limited by any written constitution, is often said by law-writers to be omnipotent. It is unnecessary to discuss here the abstract question, which cannot affect the purpose of this article, which has been sometimes suggested, whether the courts would have the right to disregard and disobey a statute passed in flagrant and outrageous violation of right and justice for that reason alone.

Every member of a political community holds his property, liberty, and life subject to the demands of the public welfare; and these demands are made and met in every State in the imposition of taxes, the exercise of the right of eminent domain, the exercise of various police powers, the raising of armies and conduct of war, and the punishment of criminals.

I have been speaking of the powers necessarily inherent in every organized political community. The limitations, which such a community may see fit to impose upon those to whom it may delegate its legislative powers, form another branch of the subject to be mentioned presently.

There is no private relation in which the State has such deep and vital stake as in the family relation. It lies at the very base of the social and political organization, and it is at once the right and the duty of the State to protect it. For this reason, as well as for the reason that the State has an interest in the health and morals of all its members, the vice of prostitution, which strikes directly at all these interests, is a proper object of legislative repression and control. The mode and extent of such repression or control is at the discretion of the legislative power.

But legislative powers in the political communities forming the American Union are limited and controlled by the Federal Constitution, and the Constitutions of the several States. For the purposes of this article it is sufficient to say that Congress possesses only such legislative powers as are expressly or impliedly conferred by the Federal Constitution—that the legislatures of the several States possess all the legislative authority inherent in their respective political communities, except as limited by the terms of the Federal Constitution, or the Constitution of the particular State.

There is nothing in any of those instruments which forbids the legislature to declare acts of illicit intercourse criminal, and to punish them accordingly, when duly proved to have been committed.

The only point of difficulty is, as to the right of the legislative power to provide for the arrest and detention of persons of either sex upon suspicion merely, and to make physical examination of their persons for the purpose of obtaining evidence of crime, or of adopting sanitary precautions or remedies.

The Constitution of the United States declares that no person shall "be deprived of life, liberty, or property, without due process of law." This provision is incorporated in similar or equivalent words, in probably all of our State Constitutions, and had its origin in Magna Charta, which, like some of the State Constitutions, uses the expression, "by the lawful judgment of his peers, or the law of the land:" *per legale judicium parium suorum, vel per legem terræ.*

Daniel Webster's explanation of this clause is often quoted: "By the law of the land is most clearly intended the general law, which hears before it condemns; which proceeds upon inquiry, and renders judgment only after trial. The meaning is that every citizen shall hold his life, liberty, property, and immunities under the protection of general rules which govern society. Everything which may pass under the form of an enactment, is not the law of the land."

The Supreme Court of New York says: "It cannot mean less than a prosecution or suit instituted and conducted according to the prescribed forms and solemnities for asserting guilt, or determining the title to property."

Under these and other constitutional provisions, persons suspected of crime may be arrested and detained for the purposes of trial; but they have the constitutional right to a speedy trial; and, in offences of this grade, to immediate and reasonable bail, until a trial shall be had. The power of those executing the law, is only to arrest, prove guilty, and to punish in the ordinary and regular course of judicial proceedings. But the right to arrest and detain exists only as incident to the right to try and punish; it goes no further than the necessity that originates it, and certainly would not include the right to compel the suspected party to submit to a physical examination, for the purpose of procuring evidence, or for any other purpose.

Such forcible examination, if not authorized by law, would be a gross assault and battery; and, in the case of a virtuous woman, would be a horrible outrage, a very few repetitions of which would produce and justify a rebellion in any community.

If a woman, or a man (for there is no difference as to the constitutional rights of the two sexes) was duly proven guilty of single or habitual acts of illicit intercourse, a physical examination might undoubtedly be compelled, either as a part of the punishment, or as a condition of release. And the law might undoubtedly provide for the detention and medical treatment of such convicts, until their physical condition should make it safe to turn them loose upon the world again. But this principle would not justify such an assault upon a person not proved to be guilty of any offence. The law takes no cognizance of offences before they are committed, and does not imprison a person to prevent the chance of his committing an offence in the future.

The law might provide that every person who should be guilty of acts of illicit intercourse, or who should make such acts a vocation, should be subjected to compulsory physical examination; but the commission of such acts, or the carrying on of such vocation, would be facts which would have to be proved in "due process of law."

The legislature may, in its discretion, license prostitution as a vocation, or provide for the registration of harlots, and may, of course, require submission to physical examination as a condition for such registration or license. A voluntary registration or acceptance of a license under such a law, would be an admission of the fact of harlotry, and a waiver of all objection to the regulations imposed as conditions.

On this principle, persons coming within the limits of a State from infected districts, may be temporarily imprisoned in quarantine; for by their own act they have consented to the imprisonment. Persons afflicted with the small-pox, or other contagious diseases, may be seized and conveyed to a pest-house for temporary safe keeping and treatment. For the going abroad among the community with such a disorder, of itself constitutes a nuisance which must be abated summarily. But the possession of a treasure of syphilis, and the transportation of it in the community, does not of itself constitute a nuisance. The owner of this wealth of infection may make it a nuisance by distributing it unlawfully, and in that case he or she may be dealt with accordingly. But the mere possession of it, without anything more, is not a nuisance or a crime; and the intent to distribute it cannot be inferred as matter of law from the mere fact of possession.

There is, therefore, no constitutional objection to statutes providing for the punishment of fornication and harlotry upon due and legal conviction; nor probably to provisions for the medical examination and treatment, for that occasion, of the persons so convicted; nor to laws for the licensing and registration of harlots, and the medical examination and treatment of those so licensed or registered.

Probably a rigid enforcement of the law upon the loose women of the city might drive many of the worse and most notorious cases into registration, but want of evidence, negligence of officers, political influence and corruption, and official connivance, would, I have no doubt, leave the majority of them unaffected.

Such as might register could undoubtedly be regulated, controlled, and treated medicinally as might be thought best; but, except as above indicated, I do not think that the right to arrest and make physical examination, or enforce medical treatment, exists. It is not the purpose of this article to discuss the question as to the best manner of dealing with this most insoluble problem—others of better opportunities, and who have bestowed more attention upon the subject, can do this better than I; but my opinion is, that the license system, as applied to our large cities, and to be enforced by our municipal authorities and political police, will be found to be not only a humbug, but a nuisance.

I have spoken mainly of the constitutional rights of the parties engaged in the business of harlotry. One word as to the rights of the citizen and property holder. A bawdy-house is a nuisance at common law, as well as in fact. It is a nuisance which affects the comfort and well being of the neighborhood in which it is located, and the value of all the neigboring property. I do not think that by licensing such a nuisance, the legislature can deprive the citizens who are injured by it, of their right to have the nuisance abated or enjoined, or to recover damages against the party maintaining it. The legislature, by granting a license, may deprive the act of keeping a bawdy-house of its criminal character, but does not thereby deprive the citizen of his remedy for the injury done to him or to his property.

